# South African Papilionoid Legumes Are Nodulated by Diverse *Burkholderia* with Unique Nodulation and Nitrogen-Fixation Loci

**DOI:** 10.1371/journal.pone.0068406

**Published:** 2013-07-11

**Authors:** Chrizelle W. Beukes, Stephanus N. Venter, Ian J. Law, Francina L. Phalane, Emma T. Steenkamp

**Affiliations:** 1 Department of Microbiology and Plant Pathology, Forestry and Agricultural Biotechnology Institute (FABI), University of Pretoria, Pretoria, Gauteng, South Africa; 2 Agricultural Research Council (ARC), Plant Protection Research Institute, Pretoria, Gauteng, South Africa; Ghent University, Belgium

## Abstract

The root-nodule bacteria of legumes endemic to the Cape Floristic Region are largely understudied, even though recent reports suggest the occurrence of nodulating *Burkholderia* species unique to the region. In this study, we considered the diversity and evolution of nodulating *Burkholderia* associated with the endemic papilionoid tribes Hypocalypteae and Podalyrieae. We identified distinct groups from verified rhizobial isolates by phylogenetic analyses of the 16S rRNA and *recA* housekeeping gene regions. In order to gain insight into the evolution of the nodulation and diazotrophy of these rhizobia we analysed the genes encoding NifH and NodA. The majority of these 69 isolates appeared to be unique, potentially representing novel species. Evidence of horizontal gene transfer determining the symbiotic ability of these Cape Floristic Region isolates indicate evolutionary origins distinct from those of nodulating *Burkholderia* from elsewhere in the world. Overall, our findings suggest that *Burkholderia* species associated with fynbos legumes are highly diverse and their symbiotic abilities have unique ancestries. It is therefore possible that the evolution of these bacteria is closely linked to the diversification and establishment of legumes characteristic of the Cape Floristic Region.

## Introduction

The rhizobial symbionts of Leguminosae indigenous to southern Africa are largely under-studied despite the significant contribution of legumes to the biodiversity of this region [1]. In the Cape Floristic Region (CFR), for example, legumes constitute about 10% of the endemic plant species [2]. Although the African continent represents one of the major centres of legume diversification [3], the root-nodule bacteria of only a few plant species have been studied. These include indigenous papilionoid legumes (e.g., *Lotononis*, *Aspalathus*, *Crotalaria*, *Desmodium* and *Alysicarpus*) that are primarily nodulated by so-called ‘alpha-rhizobia’ within the class Alphaproteobacteria [4]–[6], as well as a few species (e.g., *Cyclopia* and *Rhynchosia*) that are nodulated by ‘beta-rhizobia’ in the class Betaproteobacteria [7]–[9].

With the increased exploration of rhizobia associated with indigenous legumes during the last decade [10], many beta-rhizobia belonging to the genus *Burkholderia* have been discovered [11]. The first identified rhizobial members of this genus were *B. tuberum* and *B. phymatum* [7], [12]. *B. tuberum* is a South African papilionoid symbiont which was thought to associate with *Aspalathus carnosa* [7], [12], although this could never be proofed and instead the bacterium was later shown to nodulate a range of *Cyclopia* species [8]. *B. phymatum* was isolated in French Guiana from the papilionoid host *Machaerium lunatum* [7], [12]. All later described root-nodule members of this genus were, however, associated with mimosoid hosts from other regions of the world [12]–[17]. Also, most of the nodulating *Burkholderia* species yet to be formally described are symbionts of *Mimosa* species [17]–[20]. As a result, the association between *Mimosa* and *Burkholderia* has received considerable attention [17], including the finding that *Burkholderia* outcompetes its alpha-rhizobial co-symbionts for nodulation of *Mimosa* under nitrogen limiting conditions [21].

The genus *Burkholderia* currently includes 66 formally described species [22]. In addition to the nine species capable of establishing nitrogen-fixing symbioses with legumes (i.e., *B. caribensis*, *B. diazotrophica, B. fungorum, B. mimosarum*, *B. nodosa*, *B. phymatum*, *B. sabiae*, *B. symbiotica*, *B. tuberum*) [12]–[14], [16], [23]–[25], the members of this genus display many unique characteristics and occupy diverse environments [26]. For example, the *B. cepacia* complex includes well-known human pathogens [27] and insect endosymbionts such as *B. cenocepacia* [26]–[28], while species like *B. bryophila* have plant growth promoting properties and antifungal characteristics [29]. This diversity in environmental associations is also reflected in the evolutionary history of the genus, where species that interact with plants in a beneficial way generally form part of a lineage that is distinct from animal/human and plant pathogenic species [17], [30], [31]. Studies pertaining to the evolution of South African nodulating species of *Burkholderia* are restricted to only one published report, which suggested that the two *Burkholderia* isolates obtained from a papilionoid host in the CFR are specifically adapted to the acid soils of this region and that they are distantly related to those species that nodulate *Mimosa* elsewhere in the world [9].

To study the diversity and evolution of rhizobia, three sets of DNA markers are commonly employed, which respectively target housekeeping, nitrogen-fixation and nodulation loci. This is because the loci determining nitrogen-fixation (*nif* and *fix*) and nodulation (*nod*) are located on mobile elements (i.e., symbiosis islands or plasmids) [32], [33] that form part of the so-called accessory component [34] of the genomes of these bacteria. In the case of nodulating *Burkholderia* these symbiosis genes have been shown to form part of a plasmid [35], [36]. Consequently, these loci and the genes located on them are predisposed to horizontal gene transfer (HGT) [37], [38]. As a result their evolutionary histories are usually markedly different from those of housekeeping genes or regions located on the core genome that are in theory less prone to HGT (e.g., [34], [39]).

To trace the evolution of nodulation in rhizobia, the gene encoding NodA acyltransferase is commonly employed [11]. This enzyme, together with NodB deacetylase and NodC N-acetylglucosaminyltransferase are responsible for the formation of the signalling molecules, termed Nod factors, needed for host infection and nodule organogenesis [40], [41]. For studying the evolution of nitrogen-fixation in *Burkholderia*, the most commonly used marker is the gene encoding NifH dinitrogenase reductase, which is required for the formation of functional nitrogenase [35]. For resolving species relationships among isolates of *Burkholderia* the genes encoding the 16S ribosomal RNA (rRNA) subunit and the RecA recombinase are most commonly used [11].

In this study we considered the origins and evolution of the rhizobial symbionts associated with 15 papilionoid legume species indigenous to the Western Cape Province (WCP) of South Africa and that form part of the sclerophyllous shrubland or ‘fynbos’ vegetation of the CFR. These legumes included the three species found in the tribe Hypocalypteae (*Hypocalyptus sophoroides*, *H. oxalidifolius* and *H. coluteoides*) as well as selected species in the related tribe Podalyrieae (*Virgilia oroboides*, *Podalyria calyptrata* and ten species of *Cyclopia*). The CFR represents the centre of diversification for both these tribes [3], which share a long and complicated taxonomic history [42], [43], as well as a range of unique genetic, physiological and morphological characters [44]. Apart from a preliminary report that *Cyclopia* species are nodulated by *Burkholderia* species and a range of alpha-rhizobia [45], very little is known about the rhizobial symbionts in these two tribes. Our specific aims were therefore two-fold: (i) to obtain from these legumes a set of rhizobial isolates with verified nodulation capabilities and (ii) to infer the evolutionary histories of these isolates by means of markers from the core and accessory components of their genomes.

## Materials and Methods

### Rhizobial Isolates

For each of the 15 species of legume included in this study (Table 1), nodules were originally obtained from either the roots of plants growing in the field, or from plants grown from seed under greenhouse conditions in soil collected from the legume rhizosphere [46]. Isolation of the majority of *Hypocalyptus* isolates were performed with the permits no. 1/2002 and no. 001/241/0043 both issued by the Western Cape Nature Conservation Board, while the bacteria from the root nodules of *Virgilia* and *Podalyria* were isolated by a member of the Agricultural Research Council’s Plant Protection Research Institute (ARC-PPRI) which at that stage had the authority to collect as long as they obtained permission from the land owner. The *Cyclopia* isolates formed part of a previous PhD study [45] and were received from the ARC-PPRI and the Botany Department of the University of Cape Town respectively. Therefore, all necessary permits were obtained for the described study, which complied with all relevant regulations. For isolation of rhizobia, root nodules were surface-sterilized by soaking in 3.5% (w/v) sodium hypochlorite for 10 to 15 min and then rinsed in five changes of sterile water. The nodules were crushed with sterile forceps onto Yeast Mannitol Agar containing Congo red (diphenyldiazo-bis-α-naphthylaminesulfonate; [46]), streaked out to obtain single colonies and incubated at 28°C for 4 days until growth started appearing. All of the isolates in this study are maintained on 20% glycerol at −70°C at the University of Pretoria and can be obtained from the South African Rhizobium Collection of the ARC-PPRI.

**Table 1:** Geographic origin and nodulation ability of the *Burkholderia* isolates associated with indigenous species in the tribes Podalyrieae and Hypocalypteae.

Legume Host^a^	Isolate^b^	Geographic Origin
*H. coluteoides*	**RAU2b** (S, C, HC); **RAU2c** (S); **RAU2d**; **RAU2d2** (S, HC); **RUA2f** (S, C);	Storms River Bridge
	**RAU2g** (S, HC); **RAU2h** *; **RAU2i** * (S, C, HC); **RAU2j** * (S, C); **RAU2k** * (S, C);	
	**RAU2l** * (S, C, HC)	
*H. oxalidifolius*	**RAU6.4a** * (S, C); **RAU6.4b** * (S, C, HC); **RAU6.4d** (S, C, HC); **RAU6.4f** (S, C);	Fernkloof Nature Reserve
	**HC6.4b** c	
*H. sophoroides*	**WK1.1a** (S, C); **WK1.1c** (C); **WK1.1d** * (S); **WK1.1e** * (S, C); **WK1.1f** * (S); **WK1.1g** * (S); (S(S);	Old du Toit’s Kloof Pass
	**WK1.1h** * (S, C); **WK1.1i** * (S, C); **WK1.1j** * (S, C); **WK1.1k** * (S, C) **WK1.1m** * (C)	
*H. sophoroides* c	**HC1.1a1**; **HC1.1a2** (C); **HC1.1a3** (S); **HC1.1ba** (S); **HC1.1bb** (S); **HC1.1bc**; **HC1.1bd**;	Old du Toit’s Kloof Pass
	**HC1.1be** (S); **HC1.1bh** (C)	
*P. calyptrata*	**WC7.3a**; **WC7.3b**; **WC7.3c** (S); **WC7.3d** (S); **WC7.3f** (S); **WC7.3g**	Paarl Rock Nature Reserve
*V. oroboides*	**Kb1A** (S); **Kb2** (S); **Kb6**; **Kb12** (S); **Kb13** (S, C); **Kb14** (S); **Kb15** (S, C); **Kb16** (S, C)	Kirstenbosch Botanical Gardens
*C. buxifolia*	**CB2**	Helderberg, Somerset-West
*C. genistoides*	**UCT2**	Rein’s Farms
*C. genistoides*	**UCT15**	Constantiaberg
*C. glabra*	**UCT34**	Matroosberg, Ceres
*C. glabra*	**UCT71**	Unknown
*C. intermedia*	**CI1**; **CI2**; **CI3**	Dennehoek, Joubertina
*C. longifolia*	**Clong1**; **Clong3**	Thornhill, Humansdorp
*C. maculata*	**CM1**	Paarlberg, Paarl
*C. maculata*	**UCT70**	Jonkershoek
*C. meyeriana*	**UCT43**; **UCT56**	Hottentots Holland mountains
*C. pubescens*	**Cpub6**	Next to N1, Port Elizabeth
*C. sessiliflora*	**Cses4**	Plattekloof, Heidelberg
*C. sessiliflora*	**UCT30**	Callie’s Farm, Heidelberg
*C. sessiliflora*	**UCT31**	Grootvadersbosch
*C. subternata*	**CS2**	Dennehoek, Joubertina

a The legume hosts are listed with abbreviated genus names, i.e., *H.* = *Hypocalyptus*; *V.* = *Virgilia*; *P.* = *Podalyria*; *C.* = *Cyclopia*.

b The name of the isolates are in bold while the letters in brackets indicate the host plants on which nodulation tests were successful. S = siratro (*Macroptilium atropurpureum*; Tribe Phaseoleae), C = cowpea (*Vigna unguiculata*; Tribe Phaseoleae), HC = *Hypocalyptus coluteoides*. No brackets indicate nodulation not confirmed, except for the *Cyclopia* isolates which are indicated as authenticated in Dr. Kock’s thesis [41].

c Isolated by author using glasshouse trapping techniques.

* These isolates were originally isolated from trapping experiments and not directly from nodules in the field.

The nodulation abilities of all the isolates obtained from the root nodules of *Virgilia* and *Podalyria* were confirmed on their respective hosts at the ARC-PPRI (J. Bloem, pers. comm.), while those from the root nodules of *Cyclopia* were confirmed previously [45]. To confirm the nodulation abilities of the isolates from *Hypocalyptus*, nodulation tests were performed on *Hypocalyptus coluteoides*, with the *H. oxalidifolius* and *H. sophoroides* isolates also being tested on *H. coluteoides* because seed was not available for these species upon completion of the trapping experiments. All of the isolates included in this study, except for those obtained from *Cyclopia* species, were also tested on cowpea (*Vigna unguiculata*) and siratro (*Macroptilium atropurpureum*).

For the nodulation tests and trapping experiments the *Hypocalyptus* seeds were treated as follows. The *H. coluteoides* and *H. oxalidifolius* seeds were scarified in concentrated sulphuric acid for 30 min, while the treatment for those of *H. sophoroides* was extended to 45 min. After rinsing the seeds in five changes of sterile water, they were imbibed in sterile water for 3 to 4 hours respectively. The swollen seeds were then germinated by incubation in the dark at 15°C for 7 days on 1.5% water agar (w/v). The siratro seeds were scarified with concentrated sulphuric acid for 15 min, while the cowpea seeds were soaked in 3.5% (w/v) sodium hypochlorite for 15 min. The seeds were then rinsed and imbibed as before and germinated by incubation at 28°C in the dark for 7 days on 1.5% water agar (w/v).

Nodulation tests were performed in Leonard jars containing sterilized playpen sand and nitrogen-free Hoagland plant growth solution [46]. Pure cultures of each rhizobial isolate were used as inoculum, which was prepared by adding two inoculation loopfuls to 4 mL sterile water. After homogenization, 1 mL of the solution was added to each of three germinated seeds planted per isolate per Leonard jar. These seedlings were grown in a glasshouse (27–28°C 14 h-day and 15°C 10 h-night temperatures) for 6 to 8 weeks in the case of *Hypocalyptus* and 4 weeks for both siratro and cowpea. Due to practical constraints, the nodulation test was only performed once for every host. Because of limited seed, the effectiveness of individual strains was evaluated by visual comparison of plants grown under N-free conditions, i.e., effectively nodulated plants had strong growth and dark green leaves whereas uninoculated or non-nodulated plants had weak growth and yellow leaves. In addition, nodule interiors were examined for production of pink-coloured leghaemoglobin, which is essential for nitrogen-fixation in legumes [46]. After harvesting the nodules, rhizobia were isolated as described above, their genomic DNA extracted and a fragment of the 16S rRNA gene region amplified, sequenced and compared to that of the original isolate to verify their identity (described below).

### DNA Extraction, PCR and Sequencing

Total DNA was extracted from bacteria grown in 5 mL Tryptone Yeast broth (5 g/L Tryptone; Oxoid, Hampshire, UK; 3 g/L Yeast Extract; Biolab; 15 g/L agar) to which 2 mL calcium chloride dihydrate solution (CaCl_2_.2H_2_O; 440 g/L) was added after sterilisation. This medium reduced the amount of exopolysaccharides produced by the bacteria. After incubation with shaking (ca. 150 rpm) for 1 to 5 days at 28°C, the cells were harvested by centrifugation. The genomic DNA extraction method of Möller et al. [47] was used with some modifications. The extraction buffer was halved and the suspensions frozen for 15 min at −70°C before a first incubation step of 1 h at 64°C. After the second incubation, 1 volume of a phenol-chloroform-isoamyl alcohol [25∶24:1] mixture was added and vortexed before phase separation by centrifugation. This step was repeated until the aqueous-organic interphase was clear. Step five of the original protocol was not followed and isopropanol precipitation by overnight incubation was followed by centrifugation (20 817 rcf) for 30 min.

To amplify the nearly-complete 16S rRNA gene (i.e., ca. 1490 base pair (bp)) we used primers 16S-F (5′ AGA GTT TGA TCC TGG CTC AG 3′) [48] and 16S-R (5′ TAC CTT GTT ACG ACT TCA CCC CA 3′) [49], while a ca. 850 bp segment of the *recA* gene was amplified with the primers BUR1 (5′ GAT CGA RAA GCA GTT CGG CAA 3′) and BUR2 (5′ TTG TCC TTG CCC TGR CCG AT 3′) [50]. To amplify a ca. 580 bp region of the *nodA* gene, we utilised the degenerate primers NodAunivF145u (5′ TGG GCS GGN GCN AGR CCB GA 3′) and NodARbrad (5′ TCA CAR CTC KGG CCC GTT CCG 3′) [7], [45]. Amplification of a ca. 770 bp portion of the *nifH* gene was achieved by utilizing primers NifHF (5′ GCG AAT CTA CGG NAA RGG NGG 3′) and NifH2R (5′ CTC CAT CGT DAT NGG NGT NGG 3′), which were designed in this study by making use of BioEdit v7.0.5.3 [51] and the available *nifH* sequences for *Burkholderia*. For the *recA* and 16S rRNA amplifications, each of the 50 µL reactions included 50 to 100 ng genomic DNA, 10 µM of the respective primers, 2.5 mM of each dNTP, 25 mM MgCl_2_ and 0.1 U/µL Super-Therm *Taq* DNA polymerase and reaction buffer (Southern Cross Biotechnology, Cape Town, SA). Similar reaction mixtures were used for *nodA* and *nifH*, except that 50 µM of the respective degenerate primers were used and amplification was achieved using 5 U/µL FastStart *Taq* DNA polymerase and reaction buffer (Roche Diagnostics, Manheim, Germany).

The 16S rRNA and *recA* PCR cycling conditions were as follows: initial denaturation for 2 min at 94°C, 30 cycles including denaturation at 94°C for 1 min, annealing at 55°C (for 16S rRNA) and 62°C (for *recA*) for 1 min, elongation at 72°C for 1 min before the final elongation step at 72°C for 1 min. For the *nodA* PCRs, the cycling conditions included an initial denaturation at 95°C for 15 min, followed by 35 cycles of denaturation for 30 sec at 94°C, annealing for 30 sec at 63.5°C, and elongation for 1 min at 72°C, followed by a final elongation step of 5 min at 72°C. The *nifH* PCR cycle conditions were: denaturation for 15 min at 95°C; followed by 35 cycles of denaturation for 1 min at 95°C, annealing at 64°C for 1 min, elongation at 72°C for 1 min, with a final elongation step of 10 min at 72°C.

The *nodA* and *nifH* products were purified with the Qiagen PCR Purification Kit (Qiagen, Hilden, Germany) and cloned using the pGEM®-T Easy Vector System (Promega Corp., Madison, WI, USA) and Library Efficiency® DH5α™ competent cells (Invitrogen, Paisley, UK). Cloned inserts were amplified directly from single colonies using primers T7 and SP6 [52], and the same reaction volumes as those used for the *recA* and 16S rRNA PCRs. However, the cycling conditions for these colony PCRs included an initial denaturation at 94°C for 2 min, followed by 30 cycles of denaturation at 94°C for 30 sec, annealing at 52°C for 30 sec and elongation at 72°C for 2 min, again with a final elongation at 72°C for 7 min.

The colony PCR products, as well as the *recA* and 16S rRNA amplicons were purified using polyethylene glycol precipitation [52]. These products were then sequenced using Applied Biosystems’ ABI PRISM BigDye Terminator v3.0 Cycle Sequencing Kit and ABI3100 Automated Capillary DNA sequencer. The cloned products were sequenced using T7 and SP6, while the 16S rRNA and *recA* sequencing reactions utilized the original primers, except that the *recA* primer BUR1 was replaced with BUR-1F-short (5′ CGA RAA GCA GTT CGG C 3′). The raw sequence files were viewed and edited (where necessary) with the software programmes BioEdit and ChromasLite v2.01 (Technelysium, Queensland, Australia). All sequences generated during this study have been submitted to the European Nucleotide Archive (http://www.ebi.ac.uk; accession numbers are listed in Table S1). To confirm the identity of all sequenced products, completed sequences were also compared to the nucleotide sequences in GenBank (National Centre for Biotechnology Information http://www.ncbi.nlm.nih.gov/) by means of *blastn* [53], [54].

### Datasets and Phylogenetic Analyses

Datasets for all four gene regions were compiled by making use of the sequences generated in this study together with those obtained from GenBank. These datasets contained all the available sequences for the type strains of the known *Burkholderia* species, as well as sequences for various *Mimosa*-nodulating *Burkholderia* strains that are yet to be described from regions such as Costa Rica, Taiwan, Australia, Venezuela, Brazil and French Guiana [11], [17]–[20], [55]. The 16S rRNA, *nodA* and *nifH* datasets also included sequences for both of the *Burkholderia* isolates obtained from the South African papilionoid legume *Rhynchosia ferulifolia* [9]. In addition, both the 16S rRNA and *nodA* datasets included a South African isolate associated with *Lessertia herbacea* [6], while the *nifH* dataset included a number of strains isolated from sugarcane in Mexico [56].

We also constructed two datasets which consisted of only the *nifH* and *nodA* sequences generated in this study to evaluate the extent to which HGT has affected the evolution of South African *Burkholderia*. A number of isolates were not included in these datasets because the appropriate product could not be obtained despite repeated attempts (i.e., *nifH* for isolates RAU2h, Kb12 and CM1 and *nodA* for isolates WK1.1e and UCT15). To determine whether these two datasets represent congruent partitions, they were subjected to the incongruence length difference (ILD) test [57] using PAUP* v4.b10 [58] and 1000 repartitions.

The 16S rRNA sequences were aligned using MAFFT (Multiple Alignment using Fast Fourier Transformation; http://align.bmr.kyushu-u.ac.jp/mafft/online/server/) [59]. The sequences for the three protein-coding genes were manually aligned based on their inferred amino sequences using BioEdit. The datasets were also individually subjected to maximum-likelihood (ML) analyses with PHYML v3 [60] and Bayesian Inference (BI) with MrBayes v3.1 [61], [62]. The ML analyses utilized the best-fit evolutionary models as indicated by jModeltest v0.1.1 [60], [63], [64]. The best model for the 16S rRNA dataset was the TIM2 “transitional” model [64]. The *nodA* dataset used the TPM3uf model [64], while both the *recA* and *nifH* datasets utilized the General Time Reversible (GTR) model [65]. The *recA*, 16S rRNA and *nodA* models included gamma (G) correction for among-site rate variation and a proportion of invariable sites (I), while the *nifH* model did not include G. In all cases, these ML parameters were also utilized for calculating bootstrap branch support (BS) values [66] using 1000 pseudoreplicates. BI analyses of the 16S rRNA, *recA*, *nodA* and *nifH* datasets all utilized GTR+I+G. BI posterior probability (PP) values were calculated after 5 000 000 generations for both the *nodA* and *nifH* datasets using burnin-values of 1000 each. For the 16S rRNA data, PP values were calculated using 20 000 000 generations and a burnin-value of 20000, while the *recA* dataset values were calculated after 15 000 000 generations and a burnin-value of 15000.

## Results

### Nodulation Capabilities

We were able to show that most of the bacterial isolates examined are capable of inducing nodules on the roots of cowpea and/or siratro (Table 1). In contrast, only two of the five isolates from *H. oxalidifolius* induced nodules on *H. coluteoides*, while none of the isolates from *H. sophoroides* could nodulate *H. coluteoides* despite the fact that 17 of the 20 isolates from *H. sophoroides* induced effective nodules on cowpea and/or siratro (Table 1). However, only five of the eleven isolates from *H. coluteoides* induced nodules on *H. coluteoides* suggesting that the artificial glasshouse conditions used for the nodulation tests on *Hypocalyptus* may not have been optimal. Nevertheless, their apparent host-incompatibility may be associated with the marked differences observed in the sequences encoding NodA (see below) of the root-nodule bacteria isolated from *H. sophoroides* and *H. coluteoides.* More extensive nodulation tests are, however, needed to determine the host range of these isolates.

In all instances, nodules appeared effective in nitrogen-fixation as was evident from their pink interiors suggestive of the production of leghaemoglobin (Fig. S1a), as well as healthy plant growth and dark green leaf colour of nodulated plants compared to non-nodulated plants (not shown). On siratro and cowpea, the various isolates induced determinate nodules with distinctively spherical shapes. On *Hypocalyptus*, the respective isolates induced nodules that were indeterminate in shape (Fig. S1b), similar to nodules observed in the field. They were typically elongated and ‘caesalpinioid’ in shape [67], as is also found in members of the Australian tribes Mirbelieae and Bossiaeeae (which are evolutionary sisters of the tribe Hypocalypteae; [68], [69]). Some nodules had an ‘astragaloid’ shape where nodules are fused and fan-shaped (Fig. S1c), as is also found in the tribe Podalyrieae [67].

### Distinct *Burkholderia* Species nodulate Legumes in the Tribes Hypocalypteae and Podalyrieae

Comparisons of the 16S rRNA gene sequences for the 69 isolates examined in this study to those in GenBank revealed that they share ≥98% sequence similarity with those of known *Burkholderia* isolates and species. Phylogenetic analysis of the 16S rRNA dataset that included representatives for all members (described and as yet undescribed) of this genus separated the taxa into multiple clusters (Fig. S2). All of the isolates from hosts in the Hypocalypteae and Podalyrieae were included in one of the clusters (A, Fig. S2) along with the type strains for most of the known nodulating *Burkholderia* and the majority of the diazotrophic species and isolates. This cluster did not include any of the known pathogens, including those in the *Burkholderia cepacia* complex (*B. ambifaria*, *B. anthina*, *B. arboris*, *B. cepacia*, *B. cenocepacia*, *B. contaminans*, *B. dolosa*, *B. diffusa*, *B. lata*, *B. latens*, *B. metallica*, *B. multivorans*, *B. pyrrocinia*, *B. seminalis*, *B. stabilis*, *B. ubonensis* and *B. vietnamiensis*) [27], [70]. Similar groupings were observed with the *recA* dataset, where the root-nodulating and diazotrophic isolates of *Burkholderia* were primarily included in a cluster separate from the one generally harbouring the pathogenic species, although the separation between the two clusters were not as clear (Fig. S3).

**Figure 1:**
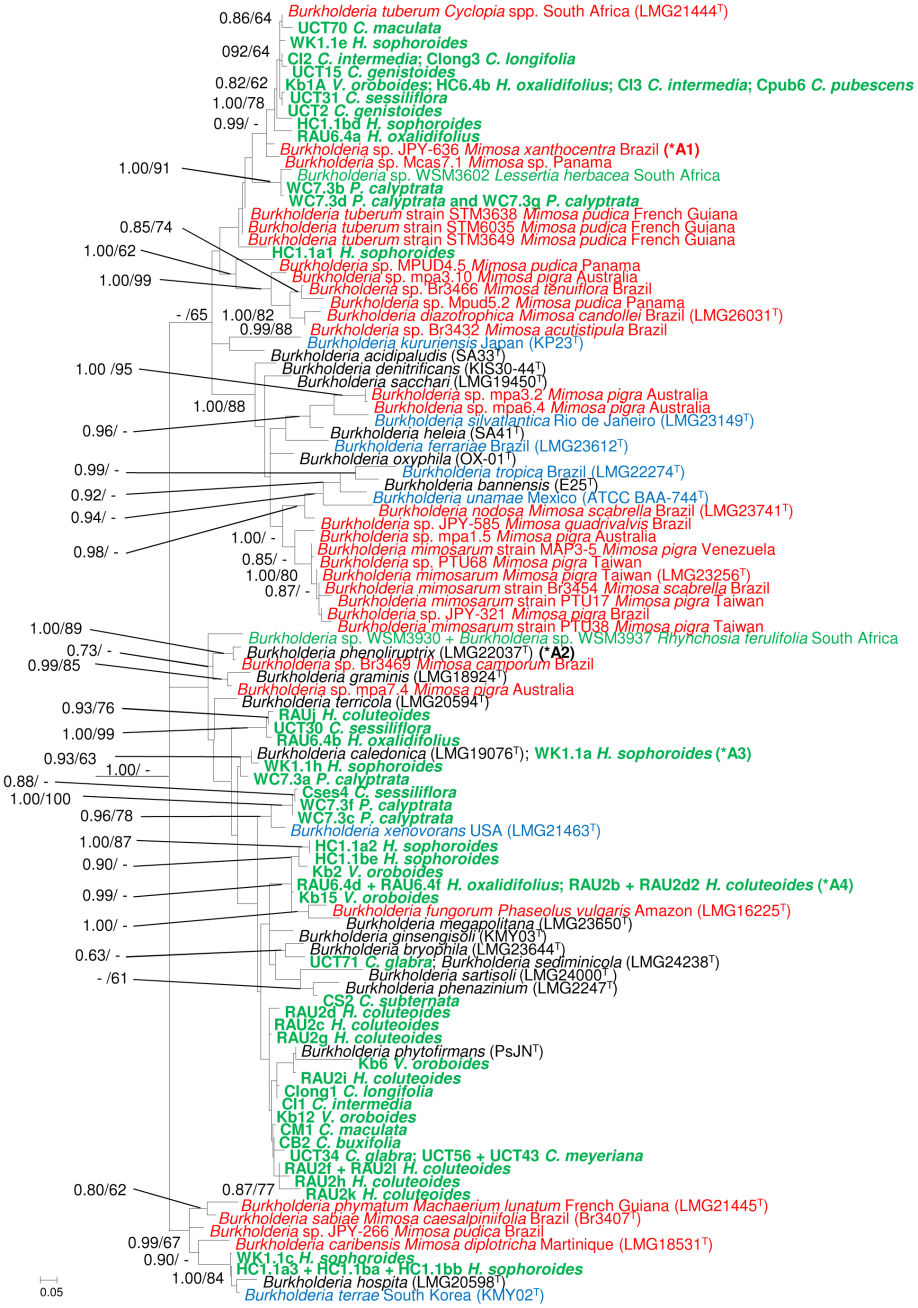
Cluster A (Fig. S2) of the 16S rRNA maximum-likelihood (ML) phylogeny for *Burkholderia*. Similar groupings were recovered following analysis of the data using Bayesian Inference (BI). Statistical support of ≥ 60% for ML bootstrap (BS) and ≥ 0.60 BI posterior probability (PP) are indicated at the branches in the order PP/BS. Diazotrophic *Burkholderia* species appear in blue, nodulating isolates in red and all nodulating South African isolates in green, with those isolates from this study also appearing in bold. All strain/isolate designations are followed by the name of the legume host and country of geographic origin. Names of the indigenous legume hosts are abbreviated as in Table 1. The GenBank accession numbers for the 16S rRNA sequences are listed in Table S1. The scale bar indicates the number of nucleotide substitutions per site. There are four groups of isolates which could not be included in full (due to size constraints) the ‘missing’ isolates are indicated as follows: *A1– *Burkholderia* sp. STM3671 *Mimosa pudica* French Guiana, *Burkholderia* sp. STM6020 *Mimosa pudica* French Guiana; *A2 - *Burkholderia* sp. JPY-582 *Mimosa hexandra* Brazil, *Burkholderia* sp. Br3462 *Mimosa flocculosa* Brazil, *Burkholderia* sp. mpa4.1 *Mimosa pigra* Australia; *A3 - WK1.1d+WK1.1f+WK1.1g+WK1.1i+WK1.1j+WK1.1k+WK1.1m+HC1.1bh *H. sophoroides* and lastly *A4 - Kb13+ Kb14+ Kb16 *V. oroboides*, HC1.1bc *H. sophoroides*.

Comparison of the 16S rRNA and *recA* phylogenies (based on 1266 and 623 aligned nucleotides, respectively), allowed us to separate the 69 isolates into consistent groups (Fig. 1 and Figs. S2 and S3). However, there was no clear trend with respect to either host or geographic locality from which the isolates originated. There were a number of groupings according to host (e.g., isolates RAU2f, RAU2l, RAU2k and RAU2h from *H. coluteoides* and isolates HC1.1be and HC1.1a2 from *H. sophoroides*), but also many groups that consisted of isolates from multiple hosts (e.g., isolates RAU6.4b, RAU2j and UCT30 from *H. oxalidifolius*, *H. coluteoides*, and *C. sessiliflora*, as well as isolates WC7.3f, WC7.3c and Cses4 from *P. calyptrata* and *C. sessiliflora*). Furthermore, some hosts were associated with multiple isolate groups. This is best exemplified by the eight isolates from *V. oroboides*, which all originated from nodules on a single tree in the Kirstenbosch Botanical Gardens, but that were mostly scattered throughout Cluster A of the 16S rRNA and *recA* phylogenies. Groupings according to location could not be readily inferred as the isolates of the majority of hosts (*H. oxalidifolius*, *H. sophoroides*, *H. coluteoides*, *P. calyptrata* and *V. oroboides*) were all collected from single localities.

In terms of the known diversity of *Burkholderia*, some of these isolates or groups of isolates were related to known nodulating or diazotrophic *Burkholderia* species (Fig. 1) (e.g., isolates UCT2, UCT15, UCT31, UCT70, WK1.1e, HC1.1bd, Kb1A, RAU6.4a, HC6.4b, CI2, CI3, Cpub6 and Clong3 are closely related to *B. tuberum*, and isolates WK1.1c, HC1.1ba, HC1.1bb and HC1.1a3 are related to *B. terrae*), while others appeared to be related to species with no known nitrogen-fixing or nodulation capabilities (e.g., isolates WK1.1a, WK1.1d, WK1.1f, WK1.1g, WK1.1i, WK1.1j, WK1.1k, WK1.1m and HC1.1bh are related to *B. caledonica* and isolate UCT71 is related to *B. sediminicola*). Overall, however, the large majority of the groups from the Hypocalypteae and Podalyrieae hosts did not group with any of the known species of *Burkholderia*.

Although we found a range of isolates that are closely related to the South African species *B. tuberum* (Fig. 1), none of our isolates associated with the two isolates (WSM3937 and WSM3930) from *Rhynchosia ferulifolia* [9]. We did however find a group of isolates from *P. calyptrata* that grouped closely with another South African isolate (WSM3602) (Fig. 1) isolated from *Lessertia herbacea* [6]. None of the other isolates originating from the root nodules of legumes in the tribes Hypocalypteae and Podalyrieae showed a close relationship to any of the undescribed *Mimosa*-nodulating strains. The only exception was isolate JPY266 [17], which formed part of a clade with moderate statistical support that also included a number of *H. sophoroides* isolates HC1.1a3, HC1.1ba, HC1.1bb and WK1.1c (Fig. 1).

### *Burkholderia* Species Associated with Hypocalypteae and Podalyrieae have Unique *nifH* and *nodA* Genes

**Figure 2:**
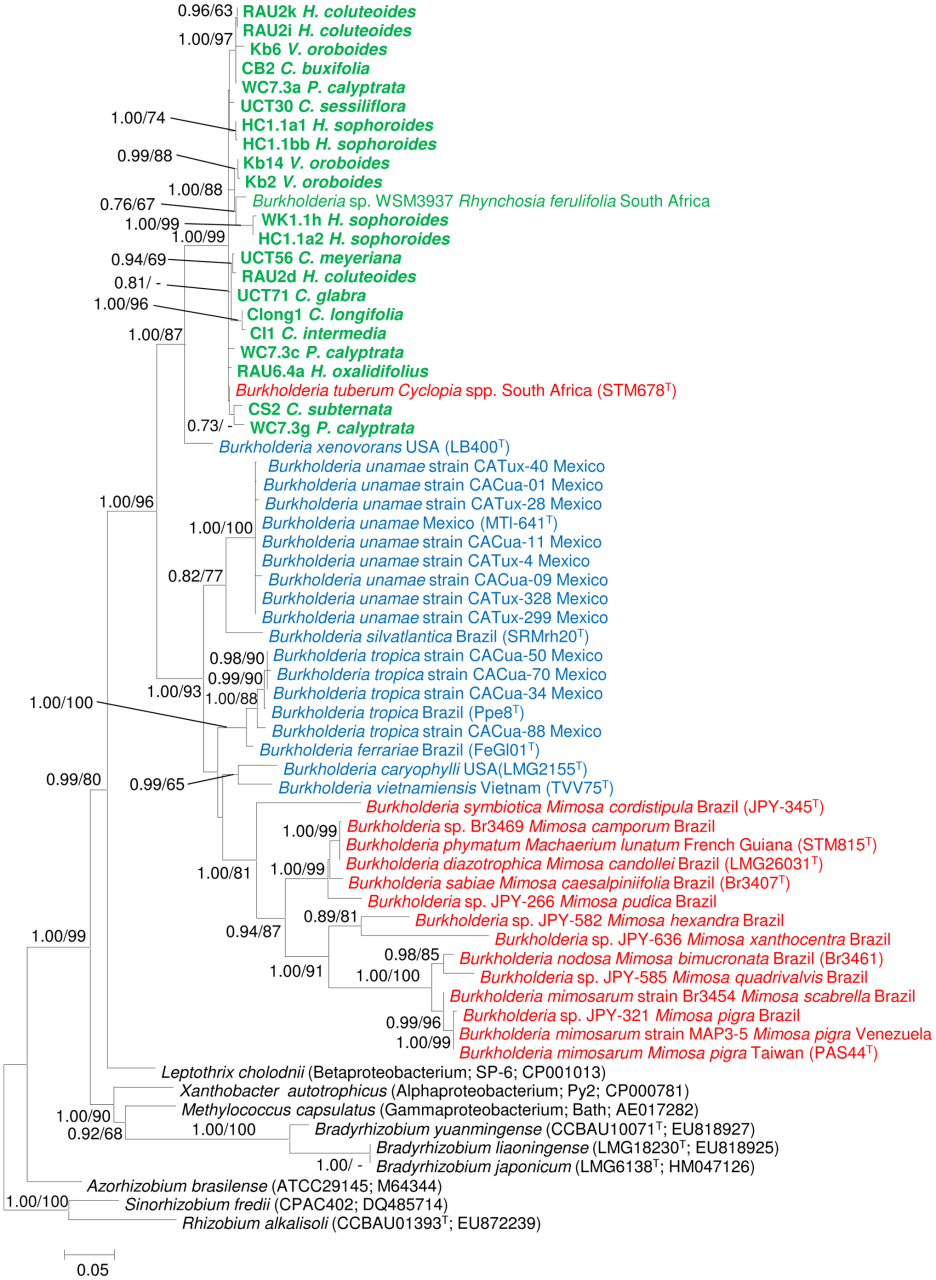
An ML phylogeny inferred from the *nifH* data for *Burkholderia.* Similar groupings were generated following BI analysis of the data. Statistical support of ≥ 60% for ML bootstrap (BS) and ≥ 0.60 BI posterior probability (PP) are indicated at the branches in the order PP/BS. Isolate/strain designations are followed by legume host names (where applicable) and geographic origin. The names of the indigenous legume hosts are abbreviated as in Table 1. Diazotrophic isolates appear in blue, nodulating isolates in red, and nodulating South African isolates in green with those from this study in bold. The GenBank accession numbers for the *nifH* sequences are listed in Table S1. The scale bar indicates the number of nucleotide substitutions per site.

**Figure 3:**
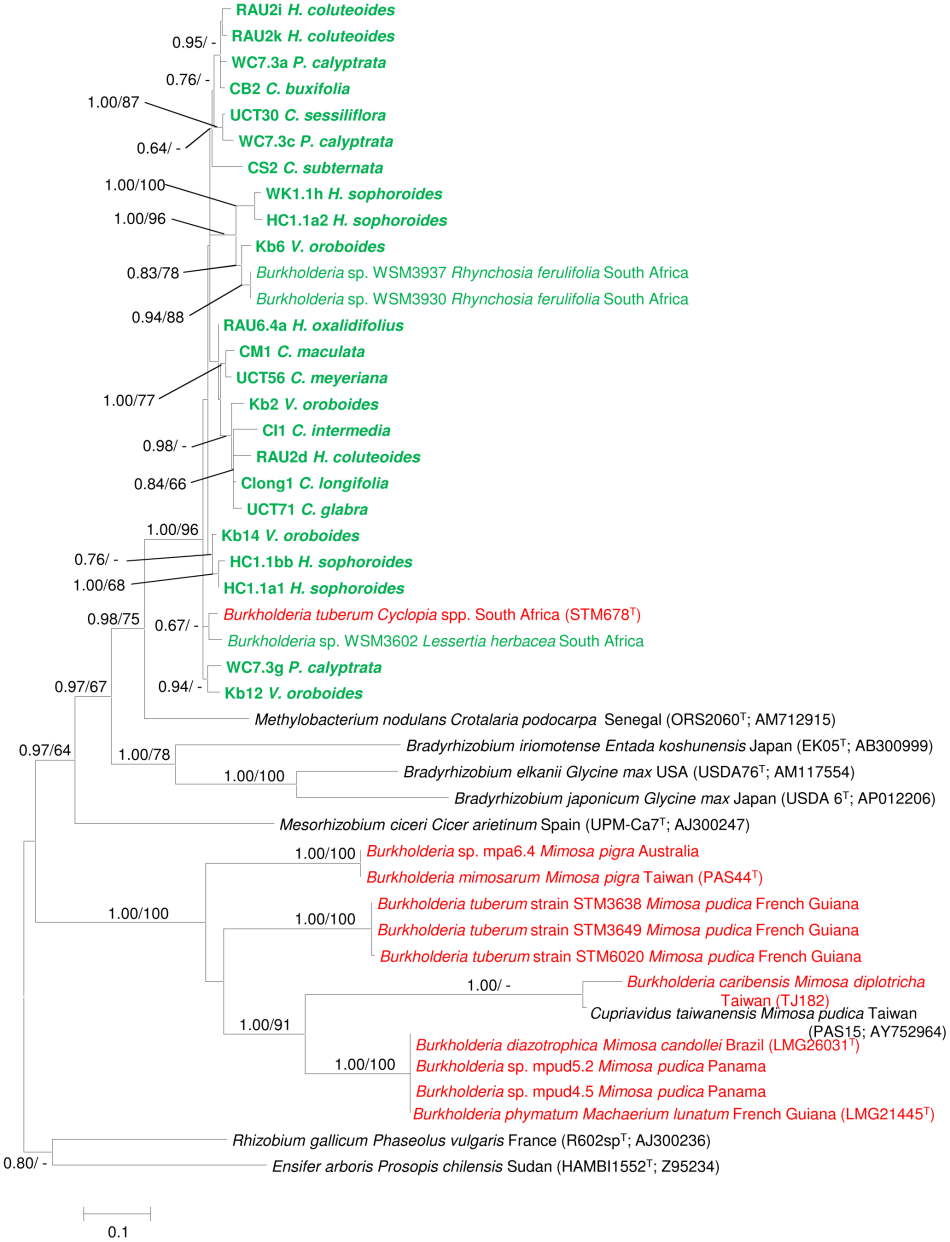
An ML phylogeny inferred from the *nodA* data for nodulating species and isolates of *Burkholderia*. Similar groupings were generated following BI analysis of the data. Statistical support of ≥ 60% for ML bootstrap (BS) and ≥ 0.60 BI posterior probability (PP) are indicated at the branches in the order PP/BS. Isolate/strain designations are followed by host name and geographic origin. The names of the indigenous legumes focused on in this study are abbreviated as in Table 1. Beta-rhizobial isolates from South Africa are indicated in green (and those from this study is in bold), while those from other regions appear in red. The scale bar indicates the number of nucleotide substitutions per site.

Comparisons of the *nifH* sequences generated in this study to those in GenBank revealed that they all shared 96–99% sequence similarity with that of isolate STM678, which is the type strain of *B. tuberum*. The *nifH* sequences of these southern African isolates showed 88–92% similarity to those of a range of nitrogen-fixing *Burkholderia* species (e.g., *B. unamae*, *B. xenovorans* and *B. tropica*). Similarly the *nodA* comparisons against GenBank showed that the sequences of our isolates were 95–100% similar to those of isolates originating from the nodules of *Cyclopia* [43], *R. ferulifolia* (strain WSM3937; [9]) and *B. tuberum* (strain DUS833; [8]). These comparisons also showed that the *nodA* sequences of southern African *Burkholderia* isolates were more similar to those of alpha-rhizobial taxa (i.e., *Methylobacterium nodulans* and *Bradyrhizobium* species) than to the *nodA* sequences of any other nodulating *Burkholderia* species or beta-rhizobia. Phylogenetic analysis of the *nifH* (Fig. 2 and Fig. S4) and *nodA* (Fig. 3 and Fig. S5) sequences further emphasized these findings, as an exclusive and well-supported assemblage containing *Burkholderia* sequences of southern African origin only was inferred from both datasets.

The *nifH* phylogeny (based on 606 aligned nucleotides) specifically included all the known sequences for diazotrophic and non-nodulating *Burkholderia* species, as well as those for representatives of free living diazotrophs in other bacterial groups (Fig. 2 and Fig. S4). Within this phylogeny, the taxon most closely related to our assemblage of South African sequences was the diazotrophic but non-nodulating species *B. xenovorans* (PP = 1.00, BS = 87%). In turn, this clade (i.e., *B. xenovorans* together with the South African *nifH* sequences) had a sister-group relationship with the well-supported clade that included various *Mimosa*-nodulating species (e.g., *B. nodosa* and *B. mimosarum*) and as yet undescribed strains, as well as various diazotrophic (but non-nodulating) species (e.g., *B. unamae*, *B. vietnamiensis* and *B. tropica*).

The *nodA* phylogeny (based on 413 aligned nucleotides) included representatives for all of the beta-rhizobia for which corresponding sequences were available, as well as for various members of the alpha-rhizobia (Fig. 3 and Fig. S5). In this tree, the taxon most closely related to the assemblage of South African sequences was the alpha-rhizobium, *Methylobacterium nodulans* (PP = 0.98, BS = 75%). This group of South African *Burkholderia* and *M. nodulans* sequences has a strongly supported (PP = 0.97, BS = 67%) sister-group relationship with an additional alpha-rhizobia clade represented by the three *Bradyrhizobium* species. The root-nodulating species and isolates of *Burkholderia* that originated from regions of the world other than southern Africa, as well as the species in another beta-rhizobial genus, *Cupriavidus*, all formed a well-supported (PP = 1.00, BS = 100%) clade distinct and not related to the South African isolates examined in this study.

Comparison of the *nifH* and *nodA* datasets that included only the sequences generated in this study revealed that they supported markedly different phylogenies (Fig. S6). For example, we found only two consistent and well-supported groupings, one of which contained 12 isolates from *H. sophoroides* (HC1.1be, HC1.1a2, HC1.1bh, WK1.1m, WK1.1i, WK1.1d, WK1.1h, WK1.1g, WK1.1k, WK1.1a, WK1.1j and WK1.1f) (*nifH* PP = 1.00, BS = 100%; *nodA* PP = 1.00, BS = 89%), and the other including four isolates from *H. coluteoides* (RAU2i, RAU2l, RAU2f and RAU2k) (*nifH* PP = 1.00, BS = 64%; *nodA* PP = 0.93). Among the collection of isolates there were several additional groups common to both phylogenies, albeit with limited statistical support. Overall, however, the relationships among groups and among numerous isolates were not similar in the two phylogenies. This was also mirrored in the ILD test results, which indicated that the *nifH* and *nodA* datasets represented non-homogenous or incongruent partitions (P value of 0.001).

## Discussion

The collection of *Burkholderia* isolates obtained from the root nodules of the *Hypocalyptus*, *Virgilia*, *Podalyria* and *Cyclopia* species investigated in this study were unexpectedly diverse. This diversity is, however, likely to represent only a small fraction of the true diversity of *Burkholderia* capable of nodulating legumes in the CFR. We included the symbionts of only 15 different legume species that were mostly sampled in single locations. In other words, our study was restricted to the root-nodule bacteria of only about 0.31% of the known legume species endemic to the CFR [2]. Nevertheless, our nodulating *Burkholderia* isolates generally spanned multiple phylogenetic groups, e.g. the eight isolates from the nodules on a single tree of *Virgilia oroboides* generally grouped with other isolates and not one another. The observed diversity of nodulating *Burkholderia* generally also appeared to be novel and mostly unrelated to the known nodulating members of this genus from other parts of the world. Our data thus suggest that detailed analyses of the rhizobial symbionts of legumes in the CFR will reveal a diversity of nodulating *Burkholderia* and other rhizobia fitting that of an internationally recognized biodiversity hotspot [71].

The distribution of nodulating *Burkholderia* species is thought to be dependent on environmental factors rather than the availability of a specific host-legume [9], [17]. Various authors suggest that soil parameters, especially pH, are important determinants of distribution [9], [11], [17], [72]. The results of recent studies indicated that a distinct assemblage of nodulating *Burkholderia* isolates is associated with Australian papilionoid legumes occurring in alkaline soils [72], [73]. In South Africa, nodulating *Burkholderia* strains have been shown to associate with legumes occurring in nutrient poor acidic soils [9], [17], [72]. Under these conditions, efficient nodulation was demonstrated for *B. tuberum* on a range of *Cyclopia* species [8] and *Burkholderia* isolates WSM3930 and WSM3937 on *Rhynchosia ferulifolia* [9]. The results of our study are thus not unexpected as nodulating *Burkholderia* species, like their legume partners, are probably well adapted to the nutrient poor and acidic soils of the CFR [8], [74], although various alpha-rhizobia apparently share this property. For example, *Lebeckia* species endemic to the CFR are known to be nodulated by species of *Mesorhizobium*, *Bradyrhizobium* and *Sinorhizobium* [75]. However, despite its likely importance, soil pH alone cannot explain the distribution of nodulating *Burkholderia* isolates [17]. In their study of the *Burkholderia* symbionts of Brazilian *Mimosa* species, Bontemps and colleagues suggested that other environmental factors such as geology and altitude, specifically the effect of the latter on temperature and/or rainfall, represent valuable predictors of the distribution of nodulating *Burkholderia* species [17].

Based on single and concatenated housekeeping gene phylogenies, the genus *Burkholderia* was divided into large clusters that generally reflect their associative environments [17], [30], [31]. This split in the genus was also observed in our 16S rRNA and *recA* phylogenies, with all of the root-nodule bacteria isolated from the *Hypocalyptus*, *Virgilia*, *Podalyria* and *Cyclopia* species investigated forming part of single cluster together with other nodulating *Burkholderia* species [12]–[14], [16]. This group also includes various diazotrophic species and species capable of associative nitrogen-fixation on non-legumes such as maize and coffee [76], [77]. The general predisposition of *Burkholderia* species to interact with plants in a positive way, especially their ability to nodulate and establish nitrogen-fixing symbioses with legumes thus appears to have arisen in the ancestor of this group. Although the exact occupancy of this group of *Burkholderia* species and isolates is still being investigated (i.e., the placement of certain species change depending on the analysis or dataset used; compare our 16S rRNA and *recA* phylogenies to published trees [17], [30], [31]), a proposal to move all of the species and isolates found in this group to a new genus *Caballeronia* is currently underway [72].

Apart from four notable exceptions, the large majority of the *Burkholderia* isolates examined here appeared to be new to science (Fig. 1). Thirteen of our isolates appeared to be conspecific to *B. tuberum*, which would expand the host range for this species to include not only *Cyclopia intermedia*, *C. pubescens*, *C. genistoides*, *C. galioides*, *C. falcata* and *Macroptilium atropurpureum* [8], but also *Cyclopia maculata*, *C. pubescens*, *C. sessiliflora*, *H. sophoroides*, *H. oxalidifolius* and *V. oroboides*. Two groups were associated with species without any known nitrogen-fixing or nodulating capabilities. The first of these is represented by a single isolate (UCT71) from *C. glabra*, which is apparently conspecific to *B. sediminicola* that was isolated from freshwater sediment in South Korea [78]. The second is represented by nine isolates (WK1.1a; WK1.1d, WK1.1f, WK1.1g, WK1.1i, WK1.1j, WK1.1k, WK1.1m and HC1.1bh) from *H. sophoroides* that appear to be conspecific to *B. caledonica* that is commonly isolated from rhizosphere soil in the United Kingdom [79]. Our data further suggests that some of our isolates (WK1.1c, HC1.1ba, HC1.1bb and HC1.1a3) associate with *B. hospita* and the diazotrophic species *B. terrae* (Fig 1). The latter species was originally isolated from forest soil in South Korea [80], while *B. hospita* was first isolated from agricultural soil in Belgium [81]. That these two known species are included in a single cluster together with four of our isolates, suggests that analysis of additional housekeeping regions is needed to fully resolve the relationships among *Burkholderia* species.

The results of this study show that HGT is an important force in the evolution of South African nodulating *Burkholderia*. Similar findings were previously demonstrated for nodulating *Burkholderia* found elsewhere in the world [11], [18], [35]. For example, our phylogenies inferred from *nifH* and *nodA* sequences were incongruent as was observed for *Burkholderia* isolates from *Mimosa* species in Taiwan, Brazil, Venezuela and French Guiana [11], [18], [35]. Furthermore, the groups recovered from the two phylogenies were not concordant with the indigenous groups supported by the 16S rRNA and *recA* data. The only exceptions were two clusters of isolates from *H. sophoroides* (HC1.1be, HC1.1a2, WK1.1m, WK1.1i, WK1.1d, HC1.1bh, WK1.1h, WK1.1g, WK1.1k, WK1.1a, WK1.1f and WK1.1j) and *H. coluteoides* (RAU2k, RAU2f, RAU2l and RAU2i), respectively, which were consistent in both *nifH* and *nodA* gene trees and, to some extent, the housekeeping gene phylogenies. The detection of such consistent groups implies that parent-to-offspring or vertical transmission of genetic information, specifically those encoding symbiotic properties, is important for some nodulating *Burkholderia*, as observed in rhizobia such as *Bradyrhizobium* [82].

Theoretically, the extent to which genetic information is shared among unrelated individuals depends largely on their spatial distribution and genetic compatibility. To determine how these factors might restrict HGT among certain groups of nodulating *Burkholderia* isolates, we examined the phylogenetic relationships among the isolates obtained from *H. sophoroides*, *H. oxalidifolius* and *H. coluteoides*. For all three species, isolates originate from plants sampled in single but distinct locations in the WCP (Table 1). Among the isolates obtained from the *H. sophoroides* and *H. coluteoides* sampling sites, we found sets of apparently unrelated isolate groups (based on housekeeping gene data) that share genotypes of *nifH* and of *nodA* that are distinct from those shared by other isolate groups in the same location (Fig. 1; Fig. S6). These distribution patterns thus indicate that genetic information is exchanged only among certain groups of *Burkholderia* in a specific location. Although there appears to be no physical barrier to HGT between the isolates, other factors such as genetic compatibility, amongst others [83], [84], might influence the rate at which successful HGT occurs.

Overall, nitrogen-fixation is thought to be a more ancient trait than nodulation and that nodulating *Burkholderia* species acquired their nitrogen-fixing abilities from free-living diazotrophic *Burkholderia* or closely related Betaproteobacteria [35], [85]. Consistent with this idea, the *nifH* sequences for all of the *Burkholderia* isolates and species included in our analysis formed an exclusive group. The observation that nodulating *B. phymatum* and *B. tuberum* are both capable of nitrogen-fixation under certain conditions, in their free-living forms, lends credibility to this hypothesis [15], [86]. However, the evolution of nodulating *Burkholderia* appears to have involved at least two independent acquisitions of nitrogen-fixation as a trait. This is evident from our *nifH* phylogeny in which all of the sequences for the South African nodulating species and isolates form a well-supported group, which is more closely related to the diazotrophic and non-nodulating species *B. xenovorans* than to other beta-rhizobia. Similarly, the nodulating species and isolates originating from elsewhere in the world form part of a second clade that also includes various non-nodulating diazotrophs. This demonstrates clearly that the nitrogen-fixing abilities of *Burkholderia* species and isolates nodulating legumes indigenous to South Africa have an evolutionary origin distinct from those of *Burkholderia* species nodulating legumes elsewhere in the world.

Comparable to the above evidence for the evolution of nitrogen-fixing capabilities, our *nodA* data indicated that the ability to nodulate legumes also developed at least twice in the genus *Burkholderia*. The *nodA* sequence-based separation of nodulating *Burkholderia* species is in agreement with previous findings [9], [11], [18], [35], [87], that the South African nodule isolates group with *Methylobacterium nodulans* (isolated in Senegal from *Crotolaria podocarpa*; [88]) and with species in the genus *Bradyrhizobium*. The phylogenetic association of nodulating South African *Burkholderia* with these alpha-rhizobia is supported by the finding that the respective *nodA* genes of *B. tuberum*, *M. nodulans* and *Bradyrhizobium* all encode a protein with an unexpectedly long N-terminal [35], [82]. In contrast, the *Burkholderia* rhizobia originating from South America and Taiwan share a common ancestor with members of the beta-rhizobial genus *Cupriavidus*, which supports previous reports [11]. A recent report suggested that the assemblage of *Burkholderia* isolates that nodulate Australian papilionoid legumes also appear to have their own distinct origin [72].

The findings presented in this study indicate a unique origin for *Burkholderia* that nodulate legumes in the CFR, especially those in the tribes Hypocalypteae and Podalyrieae. In part, this is reflected by the large diversity of *Burkholderia* isolates identified, of which a significant proportion appeared to be new to Science and only distantly related to the nodulating *Burkholderia* from other parts of the world. The uniqueness of the *Burkholderia* isolates examined in this study is, however, best exemplified by the *nodA* and *nifH* genotypes they encode as both have evolutionary origins that are distinctly different from those of non-CFR species. Our data thus suggests that the ancestry of nodulating *Burkholderia* in this region involved initial horizontal acquisition of the genetic determinants of diazotrophy, probably from indigenous free-living nitrogen-fixing *Burkholderia* species. This was followed by the acquisition of *nod* genes, probably from indigenous rhizobial symbiont(s) in the class Alphaproteobacteria. The extant products of these evolutionary processes are *Burkholderia* species that are well-adapted to the environmental conditions of the CFR (particularly its nutrient poor acid soils) and that are capable of establishing nitrogen-fixing symbioses with its fynbos legumes. It is therefore conceivable that the emergence of nodulating *Burkholderia* played an important part during radiation and establishment of the legume diversity characteristic of the CFR.

## Supporting Information

Figure S1 Nodules induced by South African *Burkholderia* isolates on the roots of *Hypocalyptus coluteoides*. **(A)** Section through an effective nodule with pink interior indicative of the presence of leghaemoglobin. **(B)** Examples of indeterminate nodules, which were observed most frequently on the roots of *H. coluteoides*. **(C)** Rare example of a fan-shaped indeterminate nodule. The scale bars represent 2 mm.
(PPTX)

Figure S2 A full 16S rRNA maximum-likelihood phylogeny of the genus *Burkholderia*. Similar groupings were recovered following analysis of the data using Bayesian Inference (BI). Statistical support of ≥ 60% for ML bootstrap (BS) and ≥ 0.60 BI posterior probability (PP) are indicated at the branches in the order PP/BS. Diazotrophic *Burkholderia* species appear in blue, nodulating isolates in red and all nodulating South African isolates in green, with those isolates from this study also appearing in bold. All strain/isolate designations are followed by the name of the legume host and country of geographic origin. The names of the indigenous hosts are abbreviated as in Table 1. The GenBank accession numbers for the 16S rRNA sequences are listed in Table S1. The scale bar indicates the number of nucleotide substitutions per site. The outgroups for this tree were *Pandoraea apista* and *Pandoraea norimbergensis*. Please note that although some isolates of *B. caribensis* and *B. fungorum* are able to nodulate certain *Mimosa* species and *Phaseolus vulgaris* respectively, the type strains has not been shown to be capable of nodulation and/or nitrogen-fixation.
(PPTX)

Figure S3 A maximum-likelihood phylogeny based on *recA* nucleotide data for the genus *Burkholderia*. Similar groupings were recovered following analysis of the data using Bayesian Inference (BI). Statistical support of ≥ 60% for ML bootstrap (BS) and ≥ 0.60 BI posterior probability (PP) are indicated at the branches in the order PP/BS. Diazotrophic *Burkholderia* species appear in blue, nodulating isolates in red and all nodulating South African isolates in green, with those isolates from this study also appearing in bold. All strain/isolate designations are followed by the name of the legume host and country of geographic origin. The names of the indigenous legumes are abbreviated as in Table 1. The GenBank accession numbers for the *recA* sequences are listed in Table S1. The only member of Cluster C, *B. glathei*, which could be included here showed inconsistent placement between the different gene trees, the same was also noticed by Estrada-de los Santos *et al.*, 2013. The scale bar indicates the number of nucleotide substitutions per site. The strains *B. mallei*, *B. thailandensis* and *B. oklahomensis* were used for outgroup purposes.
(PPTX)

Figure S4 A maximum-likelihood phylogeny based on *nifH* nucleotide data for the *Burkholderia* isolates of this study. Similar groupings were generated following BI analysis of the data. Statistical support of ≥ 60% for ML bootstrap (BS) and ≥ 0.60 BI posterior probability (PP) are indicated at the branches in the order PP/BS. Isolate/strain designations are followed by legume host names (where applicable) and geographic origin. Indigenous legume host names are abbreviated as in Table 1. Diazotrophic isolates appear in blue, nodulating isolates in red, and nodulating South African isolates in green with those from this study in bold. The GenBank accession numbers for the *nifH* sequences are listed in Table S1. The scale bar indicates the number of nucleotide substitutions per site. The outgroups used in this phylogeny are *Rhizobium alkalisoli* and *Sinorhizobium fredii*. For this maximum-likelihood phylogeny based on the GTR+I+G model, similar groupings were obtained following Bayesian Inferences analysis of the data (i.e., model = GTR+I+G; generations = 5 000 000; burnin = 2000). The scale bar indicates the number of nucleotide substitution per site.
(PPTX)

Figure S5 A maximum-likelihood phylogeny based on *nodA* nucleotide data for *Burkholderia* isolates included in this study. Similar groupings were generated following BI analysis of the data. Statistical support of ≥ 60% for ML bootstrap (BS) and ≥ 0.60 BI posterior probability (PP) are indicated at the branches in the order PP/BS. Isolate/strain designations are followed by host name and geographic origin. Indigenous host names are abbreviated as explained in Table 1. Beta-rhizobial isolates from South Africa are indicated in green (and those from this study is in bold), while those from other regions appear in red. The scale bar indicates the number of nucleotide substitutions per site. The tree is rooted to the outgroup taxa *Rhizobium gallicum* and *Ensifer arboris*. The evolutionary model used for this dataset is GTR+I+G. Similar groupings were obtained using Bayesian Inferences analysis of the data (i.e., model = GTR+I; generations = 5 000 000; burnin = 2000).
(PPTX)

Figure S6 Maximum-likelihood phylogenies for *Burkholderia* isolates from this study based on *nifH* and *nodA* nucleotide data. The isolate designations are followed by the name of the original legume host (names abbreviated as explained in Table 1). The *Hypocalyptus* isolates are indicated in different colours; *H. coluteoides* in pink, *H. sophoroides* in yellow and *H. oxalidifolius* in green. This is to highlight the fact that the groupings between the two phylogenies are explained by both horizontal and vertical transfer. Maximum-likelihood analysis of this *nodA* dataset used the HKY+I+G model, while the *nifH* dataset used the TIM3+G model. Similar groupings were obtained following Bayesian Inferences analysis of the *nodA* data (i.e., model = HKY; generations = 2 000 000; burnin = 50) and the *nifH* data (i.e., model = HKY; generations = 1 500 000; burnin = 40). Only bootstrap support values of ≥ 60% and Bayesian posterior probabilities of ≥ 0.60 are shown in the order PP/BS. Scale bars indicate the number of substitutions per site.
(PPTX)

Table S1 GenBank accession numbers for all *Burkholderia* species and isolates used in this study. (Beukes et al.; South African papilionoid legumes are nodulated by diverse *Burkholderia* with unique nodulation and nitrogen-fixation loci).
(DOC)
