# Supplementary material for: South African Papilionoid Legumes Are Nodulated by Diverse Burkholderia with Unique Nodulation and Nitrogen-Fixation Loci
Source: PLoS One. 2013 Jul 11;8(7):e68406. doi: 10.1371/journal.pone.0068406 (PMC3708930; doi:10.1371/journal.pone.0068406)
Supplement: Table S1 — GenBank accession numbers for all Burkholderia species and isolates used in this study. (Beukes et al.; South African papilionoid legumes are nodulated by diverse Burkholderia with unique nodulation and nitrogen-fixation loci). (DOC) [file pone.0068406.s007.doc]

**Supporting Table 1: GenBank accession numbers for all *Burkholderia* species and isolates used in this study.**

(Beukes et al.; South African papilionoid legumes are nodulated by diverse *Burkholderia* with unique nodulation and nitrogen-fixation loci)

| **Isolate** | **16S rRNA** | ***recA*** | ***nifH*** | ***nodA*** |
| --- | --- | --- | --- | --- |
| *B. acidipaludis* | AB513180 | - | - | - |
| *B. ambifaria* | AF043302 | AF323985 | - | - |
| *B. andropogonis* | X67037 | - | - | - |
| *B. anthina* | JX986972 | AF456059 | - | - |
| *B. arboris* | AM747630 | AM748095 | - | - |
| *B. bannensis* | AB561874 | - | - | - |
| *B. bryophila* | AM489501 | HQ398574 | - | - |
| *B. caledonica* | AF215704 | AY619669 | - | - |
| *B. caribensis* | Y17009 | AY644639 | - | AJ505309 |
| *B. caryophylli* | AB021423 | AY619663 | EF158806 | - |
| *B. cenocepacia* | AF148556 | AY951880 | - | - |
| *B. cepacia* | U96927 | AF143786 | - | - |
| *B. contaminans* | JX986975 | - | - | - |
| *B. denitrificans* | GU171384 | - | - | - |
| *B. diazotrophica* | HM366717 | FN543898 | FN544034 | FN908414* |
| *B. diffusa* | AM747629 | AM748103 | - | - |
| *B. dolosa* | JX986970 | AF323971 | - | - |
| *B. endofungorum* | AM420302 | - | - | - |
| *B. ferrariae* | DQ514537 | HQ398577 | EF158799 | - |
| *B. fungorum* | AF215705 | AJ549505 | - | - |
| *B. ginsengisoli* | AB201286 | - | - | - |
| *B. gladioli* | EU024168 | AY619665 | - | - |
| *B. glathei* | U96935 | AY619666 | - | - |
| *B. glumae* | U96931 | AJ551324 | - | - |
| *B. graminis* | U96939 | AY619653 | - | - |
| *B. heleia* | AB495123 | - | - | - |
| *B. hospita* | AY040365 | FJ958192 | - | - |
| *B. kururiensis* | AB024310 | AY619654 | - | - |
| *B. lata* | CP000150 | - | - | - |
| *B. latens* | AM747628 | AM922300 | - | - |
| *B. mallei* | AF110188 | CP000010 | - | - |
| *B. megapolitana* | AM489502 | HQ398583 | - | - |
| *B. metallica* | AM747632 | - | - | - |
| *B. mimosarum* | AY752958 | EU294396 | AY883420 | EU434822 |
| *B. multivorans* | Y18703 | NZ_ALIW01000510 | - | - |
| *B. nodosa* | AY773189 | EU294398 | AY533866* | - |
| *B. oklahomensis* | DQ108388 | NZ_ABBG01000052 | - | - |
| *B. oxyphila* | AB488693 | - | - | - |
| *B. phenazinium* | U96936 | AY619668 | - | - |
| *B. phenoliruptrix* | AY435213 | HQ398589 | - | - |
| *B. phymatum* | AJ302312 | AY644640 | NC_010627 | AJ505318 |
| *B. phytofirmans* | AY497470 | CP001052 | - | - |
| *B. plantarii* | U96933 | AJ551323 | - | - |
| *B. pseudomallei* | DQ108392 | - | - | - |
| *B. pyrrocinia* | AB021369 | HQ398568 | - | - |
| *B. rhizoxinica* | AJ938142 | FR687359 | - | - |
| *B. sabiae* | AY773186 | EU294397 | AY533867 | - |
| *B. sacchari* | AF263278 | HQ398592 | - | - |
| *B. sartisoli* | AF061872 | HQ398593 | - | - |
| *B. sediminicola* | EU035613 | HQ398594 | - | - |
| *B. seminalis* | AM747631 | AM748102 | - | - |
| *B. silvatlantica* | AY965240 | - | EF158807 | - |
| *B. soli* | DQ465451 | - | - | - |
| *B. sordidicola* | AF512827 | - | - | - |
| *B. stabilis* | AF097533 | AF456031 | - | - |
| *B. symbiotica* | HM357233 | FN543850 | FN543990 | - |
| *B. terrae* | AB201285 | - | - | - |
| *B. terricola* | AY040362 | AY619672 | - | - |
| *B. thailandensis* | U91838 | NC_007651 | - | - |
| *B. tropica* | AJ420332 | HQ398600 | EF158801 | - |
| *B. tuberum* | AJ302311 | AY644642 | AJ302315 | AJ302321 |
| *B. ubonensis* | AB030584 | - | - | - |
| *B. unamae* | AY221956 | - | EF158804 | - |
| *B. vietnamiensis* | AF097534 | AF143793 | EF158810 | - |
| *B. xenovorans* | U86373 | NC_007951 | CP000271 | - |
| *B. zhejiangensis* | HM802212 | - | - | - |
| Br3462 | AY773193 | - | - | - |
| Br3469 | AY773197 | - | AY533868 | - |
| Mcas7.1 | AY528706 | - | - | - |
| MPUD4.5 | AY528708 | - | - | JQ309910 |
| Br3466 | AY773195 | - | - | - |
| Br3432 | AY773188 | - | - | - |
| Mpud5.2 | AY528709 | - | - | JQ309911 |
| *B.* *mimosarum* strain MAP3-5 | AY533859 | - | AY533864 | - |
| PTU68 | AY752952 | - | - | - |
| *B.* *mimosarum* strain PTU38 | AY752956 | - | - | - |
| *B.* *mimosarum* strain PTU17 | AY752954 | - | - | - |
| *B. mimosarum* strain Br3454 | AY773191 | HQ398585 | AY533865 | - |
| mpa1.5 | DQ156088 | - | - | - |
| mpa3.2 | DQ156081 | - | - | - |
| mpa3.10 | DQ156084 | - | - | - |
| mpa4.1 | DQ156085 | - | - | - |
| mpa6.4 | DQ156082 | - | - | EU420074 |
| mpa7.4 | DQ156089 | - | - | - |
| WSM3930 | EU219864 | - | - | EU219866 |
| WSM3937 | EU219865 | - | EU219869 | EU219867 |
| WSM3602 | JN544916 | - | - | JN375837 |
| *B.* *tuberum* strain STM3638 | FN908407 | - | - | FN908420 |
| *B.* *tuberum* strain STM3649 | FN908403 | - | - | FN908415 |
| *B.* *tuberum* strain STM3671 | FN908404 | - | - | - |
| *B. tuberum* strain STM6020 | FN908405 | - | - | FN908416 |
| *B. tuberum* strain STM6035 | FN908406 | - | - | - |
| JPY-266 | FN543671 | FN543814 | FN543954 | - |
| JPY-321 | FN543702 | FN543845 | FN543985 | - |
| JPY-582 | FN543775 | FN543918 | FN544053 | - |
| JPY-585 | FN543776 | FN543919 | FN544054 | - |
| JPY-636 | FN543786 | FN543929 | FN544063 | - |
| *B. unamae* strain CATux-28 | - | - | HQ115046 | - |
| *B.* *unamae* strain CACua-01 | - | - | HQ115053 | - |
| *B.* *unamae* strain CACua-11 | - | - | HQ115055 | - |
| *B. unamae* strain CACua-09 | - | - | HQ115054 | - |
| *B. unamae* strain CATux-4 | - | - | HQ115045 | - |
| *B.* *unamae* strain CATux-328 | - | - | HQ115051 | - |
| *B. unamae* strain CATux-299 | - | - | HQ115050 | - |
| *B.* *unamae* strain CATux-40 | - | - | HQ115047 | - |
| *B.* *tropica* strain CACua-70 | - | - | HQ115058 | - |
| *B.* *tropica* strain CACua-50 | - | - | HQ115057 | - |
| *B.* *tropica* strain CACua-34 | - | - | HQ115056 | - |
| *B.* *tropica* strain CACua-88 | - | - | HQ115059 | - |
| RAU6.4a | HF674669 | HF544367 | HF544434 | HF674494 |
| RAU6.4b | HF674670 | HF544368 | HF544435 | HF674495 |
| RAU6.4d | HF674671 | HF544365 | HF544436 | HF674496 |
| RAU6.4f | HF674672 | HF544366 | HF544437 | HF674497 |
| RAU2b | HF674673 | HF544369 | HF544438 | HF674463 |
| RAU2c | HF674674 | HF544370 | HF544439 | HF674464 |
| RAU2d | HF674675 | HF544371 | HF544440 | HF674465 |
| RAU2d2 | HF674676 | HF544372 | HF544441 | HF674466 |
| RAU2f | HF674677 | HF544373 | HF544442 | HF674467 |
| RAU2g | HF674678 | HF544374 | HF544443 | HF674468 |
| RAU2h | HF674679 | HF544375 | NI | HF674469 |
| RAU2i | HF674680 | HF544376 | HF544444 | HF674470 |
| RAU2j | HF674681 | HF544377 | HF544445 | HF674471 |
| RAU2k | HF674682 | HF544378 | HF544446 | HF674472 |
| RAU2l | HF674683 | HF544379 | HF544447 | HF674473 |
| WK1.1a | HF674684 | HF544380 | HF544458 | HF674474 |
| WK1.1c | HF674685 | HF544381 | HF544459 | HF674475 |
| WK1.1d | HF674686 | HF544382 | HF544460 | HF674476 |
| WK1.1e | HF674687 | HF544383 | HF544461 | NI |
| WK1.1f | HF674688 | HF544384 | HF544462 | HF674477 |
| WK1.1g | HF674689 | HF544385 | HF544463 | HF674478 |
| WK1.1h | HF674690 | HF544386 | HF544464 | HF674479 |
| WK1.1i | HF674691 | HF544387 | HF544465 | HF674480 |
| WK1.1j | HF674692 | HF544388 | HF544466 | HF674481 |
| WK1.1k | HF674693 | HF544389 | HF544467 | HF674482 |
| WK1.1m | HF674694 | HF544390 | HF544468 | HF674483 |
| WC7.3a | HF674695 | HF544391 | HF544476 | HF674506 |
| WC7.3b | HF674696 | HF544392 | HF544477 | HF674507 |
| WC7.3c | HF674697 | HF544393 | HF544478 | HF674508 |
| WC7.3d | HF674698 | HF544394 | HF544479 | HF674509 |
| WC7.3f | HF674699 | HF544395 | HF544480 | HF674510 |
| WC7.3g | HF674700 | HF544396 | HF544481 | HF674511 |
| Kb1A | HF674701 | HF544397 | HF544469 | HF674498 |
| Kb2 | HF674702 | HF544398 | HF544470 | HF674499 |
| Kb6 | HF674703 | HF544399 | HF544471 | HF674500 |
| Kb12 | HF674704 | HF544400 | NI | HF674501 |
| Kb13 | HF674705 | HF544401 | HF544472 | HF674502 |
| Kb14 | HF674706 | HF544402 | HF544473 | HF674503 |
| Kb15 | HF674707 | HF544403 | HF544474 | HF674504 |
| Kb16 | HF674708 | HF544404 | HF544475 | HF674505 |
| HC1.1a1 | HF674709 | HF544405 | HF544448 | HF674484 |
| HC1.1a2 | HF674710 | HF544406 | HF544449 | HF674485 |
| HC1.1a3 | HF674711 | HF544407 | HF544450 | HF674486 |
| HC1.1ba | HF674712 | HF544408 | HF544451 | HF674487 |
| HC1.1bb | HF674713 | HF544409 | HF544452 | HF674488 |
| HC1.1bc | HF674714 | HF544410 | HF544453 | HF674489 |
| HC1.1bd | HF674715 | HF544411 | HF544454 | HF674490 |
| HC1.1be | HF674716 | HF544412 | HF544455 | HF674491 |
| HC1.1bh | HF674717 | HF544413 | HF544456 | HF674492 |
| HC6.4b | HF674718 | HF544414 | HF544457 | HF674493 |
| CB2 | AY178059 | HF544415 | HF544482 | HF674512 |
| UCT2 | AY178073 | HF544416 | HF544491 | HF674513 |
| UCT15 | AY178068 | HF544417 | HF544490 | NI |
| UCT34 | AY178056 | HF544418 | HF544493 | HF674514 |
| UCT71 | AY178064 | HF544419 | HF544495 | HF674515 |
| CI1 | AY178060 | HF544420 | HF544484 | HF674516 |
| CI2 | AY178069 | HF544421 | HF544485 | HF674517 |
| CI3 | AY178072 | HF544422 | HF544486 | HF674518 |
| Clong1 | AY178061 | HF544423 | HF544487 | HF674519 |
| Clong3 | AY178070 | HF544424 | HF544488 | HF674520 |
| CM1 | AY178058 | HF544425 | NI | HF674521 |
| UCT70 | AY178075 | HF544426 | HF544494 | HF674522 |
| UCT43 | AY178055 | HF544427 | HF544498 | HF674523 |
| UCT56 | AY178054 | HF544428 | HF544499 | HF674524 |
| Cpub6 | AY178071 | HF544429 | HF544483 | HF674525 |
| Cses4 | AY178063 | HF544430 | HF544489 | HF674526 |
| UCT30 | AY178067 | HF544431 | HF544497 | HF674527 |
| UCT31 | AY178074 | HF544432 | HF544492 | HF674528 |
| CS2 | AY178065 | HF544433 | HF544496 | HF674529 |

NI Sequences not included in corresponding dataset.

*Accession numbers indicated are not those of the type strains. The *nifH* sequence of *B. diazotrophica* is that of strain STM4206, while the *nodA* sequence of *B. nodosa* is that of strain Br3461.

- No sequence for the isolate appear in the dataset due to unavailability or because the available sequence was too short.
